# Whole-Genome Characterization of SARS-CoV-2 Reveals Simultaneous Circulation of Three Variants and a Putative Recombination (20B/20H) in Pets, Brazzaville, Republic of the Congo

**DOI:** 10.3390/v15040933

**Published:** 2023-04-09

**Authors:** Léadisaelle Hosanna Lenguiya, Matthieu Fritz, Daphné de Riols De Fonclare, Sandrine Corbet, Pierre Becquart, Christophe Mbou, Ruben Junias Nguie, Wivine Salva Mouellet, Jordy Exaucé Lyelet Demboux, N’kaya-Tobi, Pembe Issamou Mayengue, Félix Koukouikila-Koussounda, Meriadeg Ar Gouilh, Eric M. Leroy, Fabien Roch Niama

**Affiliations:** 1 Faculté des Sciences et Techniques, Université Marien Ngouabi, Brazzaville BP69, Congo; 2 Institut de Recherche pour le Développement, Unité Mixte de Recherche Mivegec, BP34090 Montpellier, France; 3 UNICAEN, Department of Viroloy, University of Rouen Normandie, Inserm Dynamicure UMR 1311, BP14000 Caen, France; 4 Direction Générale de l’Elevage, Brazzaville BP2354, Congo; 5 Laboratoire National de Santé Publique, Brazzaville BP120, Congo

**Keywords:** SARS-CoV-2, pets, cats, dogs, RT-qPCR, serology, NGS, Republic of the Congo (RoC)

## Abstract

Following the emergence of SARS-CoV-2, cases of pets infected with variants circulating among humans were reported. In order to evaluate the occurrence of SARS-CoV-2 circulation among pets in the Republic of the Congo, we conducted a ten-month study of dogs and cats living in COVID-19-positive households in Brazzaville and neighboring localities. Real-time PCR and the Luminex platform were used to detect SARS-CoV-2 RNA and antibodies to SARS-CoV-2 RBD and S proteins, respectively. Our results show for the first time the simultaneous circulation of several variants of SARS-CoV-2, including viruses from clades 20A and 20H and a putative recombinant variant between viruses from clades 20B and 20H. We found a high seroprevalence of 38.6%, with 14% of tested pets positive for SARS-CoV-2 RNA. Thirty-four percent of infected pets developed mild clinical signs, including respiratory and digestive signs, and shed the virus for about one day to two weeks. These results highlight the potential risk of SARS-CoV-2 interspecies transmission and the benefits of a “One Health” approach that includes SARS-CoV-2 diagnosis and surveillance of viral diversity in pets. This approach aims to prevent transmission to surrounding wildlife as well as spillback to humans.

## 1. Introduction

Since its human origin in 2019, Severe Acute Respiratory Syndrome Coronavirus-2 (SARS-CoV-2) has rapidly spread throughout the world—a global pandemic affecting 247 territories and regions worldwide [1].

In the Republic of the Congo (RoC), the first human COVID-19 case was officially reported on 14 March 2020 [2], although our group’s recent study suggests an earlier arrival in late 2019 [3]. As of January 2022, the country recorded four waves of infections in July 2020, April 2021, October 2021, and December 2021 [4]. By December 2022, the cumulative number of infections in the RoC was 24,835, with 386 deaths. More than half of these cases were reported in Brazzaville, the capital [5].

As the pandemic grew and spread from its origin in December 2019, new mutants were detected; some were subsequently labelled as “variants of concern” (VOC), “variants of interest” (VOI), or “variants under monitoring” (VUM), due to their genome variability, transmissibility, or the disease outcome [6]. Between December 2020 and November 2021, five VOCs, namely alpha, beta, delta, gamma, and omicron, became widely distributed and caused waves in several countries [7,8,9].

As SARS-CoV-2 continued its circulation and spread in humans, the first SARS-CoV-2 infections in pets were officially reported in February 2020 [10]. Presently, multiple cases of natural SARS-CoV-2 infection in pets living with infected people have been reported [11], and, as might be expected, infection rates were higher for pets living with COVID-19 infected people than for those living with people of unknown status [12,13]. Studies detecting SARS-CoV-2 RNA in pets in the USA, Thailand, and Brazil have reported variable infection rates ranging from 5.3% to 33.3% [14,15,16,17]. Pets were found positive up to one month after their owner’s positive test and were reported capable of shedding virus for more than one week. Reported seroprevalences in pets were also highly variable from one study to another, ranging from 7.8 to 44.7% [12,13,14,16,18,19,20].

While most pets affected by SARS-CoV-2 appear asymptomatic, some animals have developed clinical signs such as fever, coughing, sneezing, breathing difficulties, lethargy, loss of appetite, anorexia, vomiting, and diarrhea [21,22,23]. In addition, pets were found to shed virus for up to one week, and cat-to-cat transmission has been observed under experimental conditions [24,25]. There is presently no evidence that pets participate in the spread and maintenance of the pandemic. However, there have been recent observations of zoonotic SARS-CoV-2 transmission from an infected cat to a human in Thailand [26] and a passive mechanical transmission from a dog to a human in China [27]. While such transmission from pets to humans or other animals is currently very rare, with the appearance of each new variant, there is the potential that this risk could change or that the clinical signs of disease in animals could also change.

In its December 2022 report, the World Organization for Animal Health (WOAH) officially reported SARS-CoV-2 infection in pets in Europe, Asia, and America but none in Africa [28]. To the best of our knowledge, there are currently no reports of pet infections with SARS-CoV-2 in Africa [11,28].

To address this gap in knowledge, we conducted a full-scale study of SARS-CoV-2 circulation in dogs and cats living in households with COVID-19-positive persons in Brazzaville and neighboring localities. Between 5 February and 6 December 2021, we collected and analyzed sequentially nasopharyngeal and rectal swabs as well as blood samples from dogs and cats living with at least one owner diagnosed with COVID-19.

## 2. Materials and Methods

### 2.1. Ethics Statement

The pets’ owners were informed about the study design and gave their verbal consent. This study was conducted with the approval of the Comité Technique de la Riposte à la Maladie à Coronavirus COVID-19 through the Centre des Operations d’Urgence en Santé Publique (COUSP).

### 2.2. Study Population

**Figure 1:**
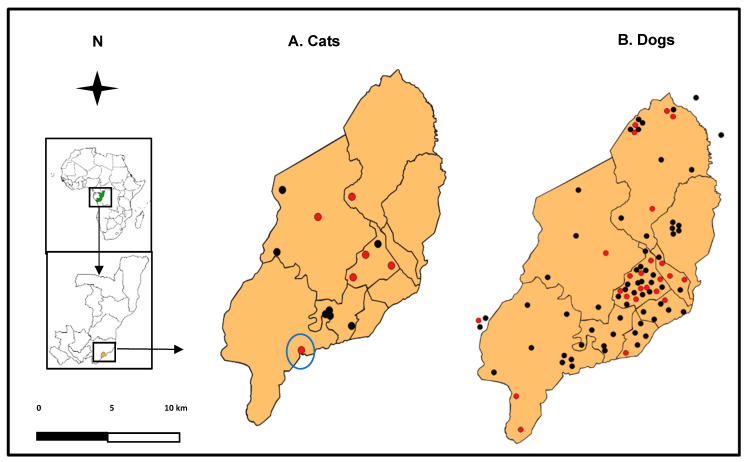
Maps of Brazzaville (RoC) showing the distribution of households where cats (**A**) and dogs (**B**) were sampled. Red and black dots represent the pets that tested positive and negative, respectively, for SARS-CoV-2. The cat with a putative recombinant variant is highlighted with a blue circle. Map data source: www.arcgis.com accessed on 3 February 2022.

The study was carried out from 5 February to 6 December 2021, in the nine districts of Brazzaville and neighboring localities, notably GOMATSETSE and IGNIE, located in the Pool department (Figure 1).

During the ten months of the study, people who had tested positive for COVID-19 were regularly contacted within two weeks of a positive test to find out if they owned pets. People that were contacted included patients with severe symptoms of the disease (respiratory difficulties, shock) at the University Hospital of Brazzaville (CHU-B), the Municipal Clinic Albert LEYONO (CMAL), the Central Hospital of the Armed Forces Pierre MOBENGO (HCAPM), the SECUREX Clinic, and the Pasteur Clinic, as well as those with mild symptoms (coughing, sneezing, fever, etc.). Asymptomatic individuals were sampled at the COUSP. Lastly, there were those tested at the National Public Health Laboratory (LNSP) or the Congolese Foundation for Medical Research (FCRM) and people screened for travel at the Maya-Maya Airport.

### 2.3. Animal Sampling

Soon after a pet owner’s COVID-19 positive test and with their consent, we visited their home to sample pets. At the home visit, we also collected data on the pet’s breed, age, sex, main clinical signs, and date of onset of clinical signs, as well as owner data, such as the date of onset of symptoms, the date of their COVID-19 positive result, and the nature of their interactions with the animal.

Animals were sampled at home with the cooperation of veterinarians under the direction of the Congolese Minister of Agriculture, Fisheries, and Livestock (MAEP). The veterinarians were provided personal protective equipment and collected two nasopharyngeal and one rectal swabs from each animal. Each nasopharyngeal and rectal swab was suspended in 500 µL of phosphate-buffered saline (PBS) in 2 mL cryotubes and placed in a coolbox until arrival at the Congolese National Public Health Laboratory (LNSP), where they were immediately analyzed or kept in a −80 °C freezer until analysis.

When possible, each animal was re-sampled two or four days after the first sampling, and animals that tested positive for SARS-CoV-2 by RT-qPCR were re-sampled until they tested negative.

From 5 August to 6 December 2021, the pet’s owners were contacted again for animal blood sampling. Blood was collected from the saphenous vein in a dry tube and transported to the LNSP, where the tubes were immediately centrifuged and sera aliquots were stored at −80 °C until analysis.

### 2.4. Molecular Analysis

#### 2.4.1. RNA Extraction

Total RNA was extracted from nasopharyngeal and rectal swabs of each pet using NucleoSpin RNA (Macherey-Nagel, GmbH & Co. KG, Düren, Germany) following the manufacturer’s recommended procedures. Briefly, nasopharyngeal and rectal samples were vigorously vortexed for 15 s, and 200 μL of the supernatant was used for the inactivation step in biological safety cabinet II. Then, the total RNA was eluted with 50 μL of RNase-free water and either immediately used for SARS-CoV-2 molecular detection or stored at −80 °C for later use.

#### 2.4.2. RT-qPCR

We performed SARS-CoV-2 molecular detection using the specific multiplex one-step RT-qPCR kit TaqPath™ COVID-19 CE-IVD (Thermofisher Scientific, Waltham, MA, USA) for amplified ORF1ab, N, and S genes of SARS-CoV-2. The run was made on an Applied Biosystems 7500 Fast thermocycler.

The generated PCR curves were evaluated using the FAST 7500 PCR visualization and interpretation software. A sample was considered positive when significant amplification—a CT value <37—was detected for at least two of the three targeted genes (Figure S1). Samples with significant amplification for only one of the targeted genes were re-assayed and declared questionably positive if the positive signal remained in the second assay.

#### 2.4.3. Genome Sequencing and Phylogenetic Analysis

We performed whole-genome sequencing of 14 SARS-CoV-2 samples using a previously described method combining an Ampliseq approach with MinION Nanopore technology, used for routine SARS-CoV2 sequencing in the virology department of the Caen University Hospital, Normandy, France [29].

Unfortunately, pet owner samples were unavailable in the LNSP for sequence comparison. Phylogenetic analyses included the 10 SARS-CoV-2 genomes of pets sequenced in this study, the reference strain NC_045512-Wuhan-Hu-1, all sequences of SARS-CoV-2 detected in humans in RoC, and representative sequences of VOCs detected in South Africa, Brazil, Botswana, and the UK available from the Global Initiative on Sharing All Influenza Data (GISAID).

SARS-CoV-2 genomes originating from Congo were extracted in January 2022 from the GISAID database and filtered on the following parameters: complete genomes, high coverage, and low coverage excluded (*n* = 89). The dataset was then aligned using MAFFT version 7.407 [30] with auto parameters and visually inspected using Seaview [30]. Phylogenetic reconstruction was performed using Beast Suite version 1.10.4 [31], using the GTR model of evolution with gamma distribution and invariable site parameters, a coalescent constant size model, collection dates as tree priors, and an uncorrelated relaxed clock model with lognormal distribution [32]. Posterior probability values were used as an estimation of node support. Four hundred million iterations of the Markov chain were launched on an 18 double-core computer in order to reach an effective sampling size over 200 for each statistic parameter. A total of ten thousand trees were computed to obtain the final maximum credibility tree.

Genomic sequences were aligned using MAFFT version 7.407 with auto parameters and visually inspected. Phylogenetic reconstruction was performed using Beast Suite version 1.10.4. We have provided the reference for our sequencing strategy and analysis pipeline [29].

### 2.5. Serological Analysis

We used a multiplex microsphere immunoassay (MIA) for the detection of immunoglobulin G (IgG) against the receptor-binding domain (RBD) and trimeric spike (tri-S) proteins of SARS-CoV-2, as described by Fritz et al. [12]. Ten µg of these two recombinant SARS-CoV-2 antigens (The Native Antigen Company, Kidlington, United Kingdom) were used to capture specific serum antibodies. Distinct MagPlex microsphere sets (Luminex Corp., Austin, TX, USA) were respectively coupled to viral antigens using the amine coupling kit (Bio-Rad Laboratories, Marnes-la-Coquette, France) according to the manufacturer’s instructions. Microsphere mixtures were successively incubated with serum samples (1:400), biotinylated protein A and biotinylated protein G (4 µg/mL each) (Thermo Fisher Scientific, Illkirch, France), and streptavidin-R-phycoerythrin (4 µg/mL) (Life Technologies, Illkirch, France) on an orbital shaker and protected from light. Measurements were performed using a Magpix instrument (Luminex Corp., Austin, TX, USA), and at least 100 events were read for each bead set. Binding events were displayed as median fluorescence intensities (MFI). In the absence of pre-pandemic serum samples from dogs and cats collected in the RoC, specific seropositivity cut-off values for each antigen were set at three standard deviations above the mean MFI of 53 dog and 30 cat serum samples collected in France before 2019. Based on the pre-pandemic population, MIA specificity was set for each antigen at 96.2% for dogs and 100% for cats.

### 2.6. Statistical Analysis

We use Fisher’s exact test to analyze differences in RT-qPCR and antibody detection in dogs and cats from COVID-19 households.

## 3. Results

### 3.1. Study Population

**Figure 2:**
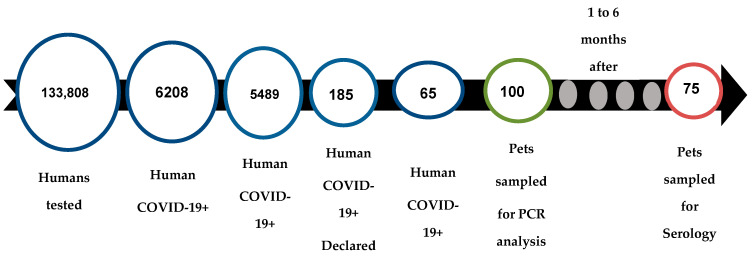
Pet’s enrolment outline.

Over the ten months of this study, 133,000 people living in the nine arrondissements of Brazzaville and its surroundings (GOMATSETSE and IGNIE) were screened for COVID-19 by the LNSP and the FCRM. In total, 6208 people were diagnosed SARS-CoV-2-positive by RT-qPCR, among whom we contacted 5483 to find out if they owned pets. Overall, 187 people indicated that they own at least one pet (dog or cat), and 65 provided consent to sample their pets (Figure 2). Among the households sampled, 72% (47/65) had one pet, and the remaining 28% (19/65) had 2 to 6 pets, representing a final sample of 86 dogs and 14 cats.

Pets were sampled from 1 to 10 days (an average of 3.9 days) after their owner’s COVID-19-positive test and from 5 to 24 days after the onset of the owner’s symptoms (Table 1). All of the pet owners reported having close contact with their pets even after the onset of their COVID-19 symptoms. Their interactions included playing, kissing, grooming, walking, and sharing food and living spaces.

Dog and cat breeds were indicated (Table S1). Pet ages ranged from 2 months to 13 years for dogs and from 2 months to 3 years for cats. The male/female sex ratio was 1.5:1 (52/34) for dogs and 0.7:1 (6/8) for cats.

### 3.2. Clinical Signs

Clinical signs were observed in 24% of pets from symptomatic and asymptomatic households. In symptomatic households, 14 dogs and 2 cats developed clinical signs within 3 to 10 days (an average of 6.85 days) after the onset of their owner’s COVID-19 symptoms. In asymptomatic households, clinical signs of infection were reported for seven dogs within two to eight days (an average of 4.5 days) prior to the owner’s COVID-19-positive test. Clinical signs included fever, fatigue, sneezing, gastroenteritis, decreased appetite and weight loss, breathing difficulties, and diarrhea. In addition, we also observed symptoms of chronic disease such as depression, mood swings, anal abscesses, wounds, cayor worms, and eczema (Table 1 and Table S1).

### 3.3. RT-qPCR Analysis

From the 100 pets in this study, nine dogs and five cats from different households tested SARS-CoV-2 positive or questionably positive by RT-qPCR (Table 1). Among the nine positive dogs, four tested positive by nasopharyngeal swabs and five by rectal swabs. For cats, all five tested positive by nasopharyngeal swabs. Interestingly, no pet tested positive for both nasopharyngeal and rectal swabs. Positive tests from nasopharyngeal swabs were observed for one cat and one dog presenting with respiratory signs (sneezing), while positive tests from rectal swabs were observed for two dogs presenting with digestive signs of infection (diarrhea or gastroenteritis).

The Ct values for positive tests ranged from 27.2 to 36.1 for dogs and 25.8 to 35.2 for cats (Table S1, Figure S1).

### 3.4. Viral Shedding Follow-Up

**Figure 3:**
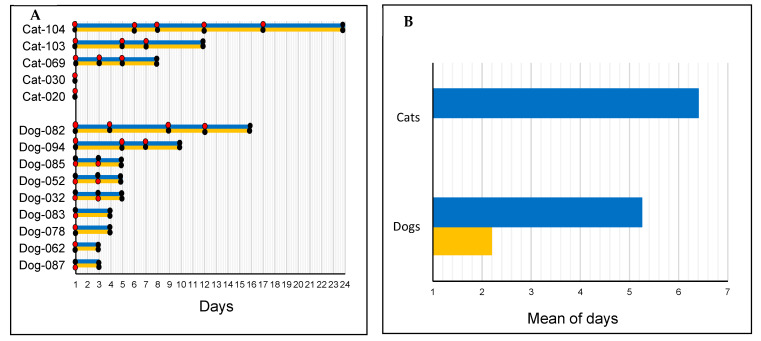
Duration of SARS-CoV-2 detection in dogs and cats from COVID-19+ households in Congo. (**A**) Duration of detection in dogs and cats. (**B**) Comparative mean duration of detection in nasopharyngeal and rectal samples from pets. Black dot—negative result; red dot—positive result; blue line—nasopharyngeal sample; and yellow line—rectal sample.

After their initial positive tests, 5 dogs and 3 cats remained SARS-CoV-2-positive in intervals of 3 to 12 and 3 to 17 days, respectively (Figure 3A). For dogs, SARS-CoV-2 detection ranged from 1 to 12 days for nasopharyngeal swabs and 1 to 3 days for rectal swabs (Figure 3B).

### 3.5. Sequencing and Phylogenetic Analysis

A total of 10 full-length SARS-CoV-2 genomes were obtained from the 14 SARS-CoV-2-positive pet samples, including sequences from 6 dogs and 4 cats. The sequences were obtained from four nasopharyngeal and two rectal samples of dogs and four nasopharyngeal samples of cats.

**Figure 4:**
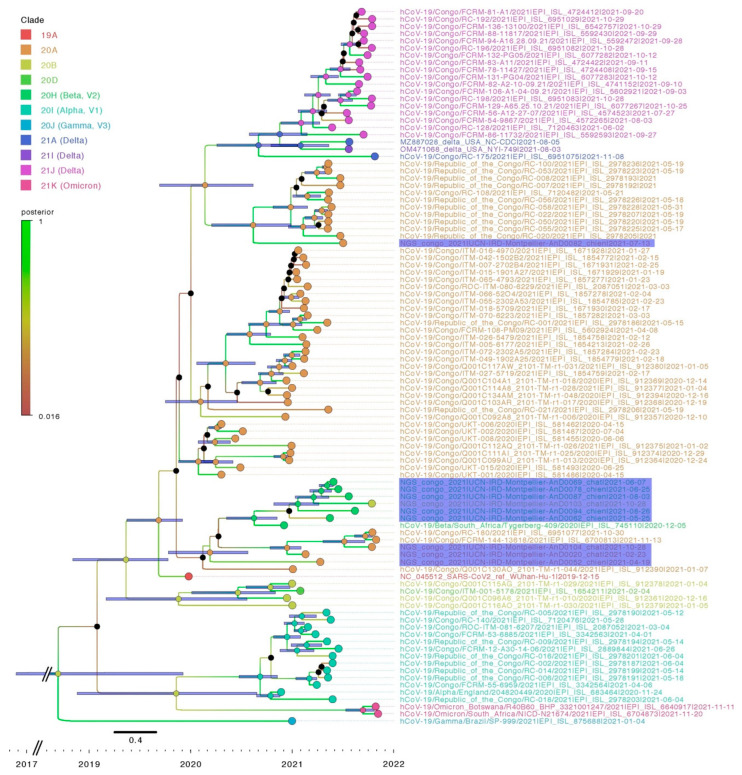
Bayesian phylogenetic analysis of ten pets’ SARS-CoV-2 whole genome sequences. The tree included eighty SARS-CoV-2 sequences from Congolese people and the reference sequences of alpha, beta, delta, and omicron VOCs available on GISAID. Pet sequences are highlighted in violet.

The comparative analysis uncovered substantial variability among pet sequences (Figure 4). Sequences in clade 20A (Pangolin lineage B1, B.1.640.1, variant) were obtained from Dog-052 and Dog-082 (sampled on 19 April 2021 and 13 July 2021, respectively), and Cat-020 and Cat-104 (sampled on 23 February 2021 and 28 October 2021, respectively). Sequences from Dog-062, Dog-078, Dog-087, Dog-94 and Cat-069, sampled between 25 June 2021 and 3 August 2021, all clustered within the 20H clade (V2, B.1351, Beta variant).

**Figure 5:**
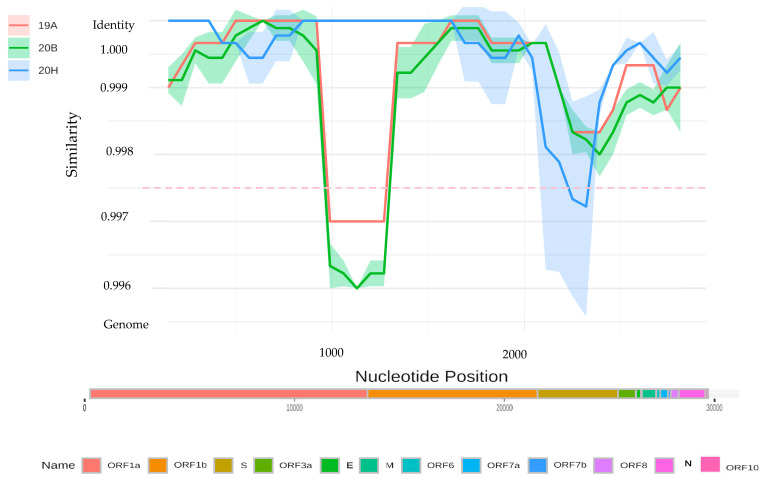
Identity plot of a putative recombinant sequence from Cat-103.

Lastly, the lineage assignment of the SARS-CoV-2 genome sequence obtained from Cat-103 proved anomalous. Nextclade analysis classified it in clade 20B (B1533 lineage), while Bayesian phylogenetic analysis positions it in clade 20H (V2, B.1351, Beta variant) (Figure 4). A sliding window analysis (comparing the identity of the Cat-103 SARS-CoV2 genome against representatives of major clades included in the phylogenetic analyses) revealed a shift in identity around the spike gene. According to this analysis, the spike gene from the Cat-103 SARS-CoV2 genome shares a greater degree of identity with the 20B clade than the remainder of the genome, which clusters within the 20H lineage (Figure 5). Recombination detection analysis performed on our samples with RDP4 [33] does not detect a significant recombination event at this position. In fact, given the high nucleotide identity between the two variants, any recombination event would be difficult to detect.

### 3.6. Serological Analysis

At the end of the study (from 9 August to 6 December 2021), sera were collected at least 1 month to 6 months after the PCR analysis. Because of death and inaccessibility to some animals, we collected blood samples from 65 (76%) dogs and 10 (71%) cats initially included in the study.

Overall, 38.6% of pets, including 22/65 (33.8%) dogs and 7/10 (70%) cats, tested positive for IgG antibodies against SARS-CoV-2 RBD and S proteins (Table S2). Among pets testing positive by MIA, 7 of the 22 dogs and 5 of the 7 cats had also tested positive by RT-qPCR (Table 2).

## 4. Discussion

This study aimed to assess the risk of SARS-CoV-2 transmission in pets living in COVID-19-positive households in the Republic of the Congo. The study occurred between 5 February and 6 December 2021, in Brazzaville and neighboring localities, during two waves of contamination in April and October 2021. This study shows for the first time a high rate of SARS-CoV-2 detection and the simultaneous circulation of multiple SARS-CoV-2 variants with a possible SARS-CoV-2 recombinant (20B/20H) in a cat.

We report a seroprevalence of 38.6% in pets, including 22 of 65 (33.8%) dogs. Although the number of cats sampled was low, 7 out of 10 (70%) were seropositive. Several studies conducted on other continents have reported variable seroprevalence rates ranging from 7.8 to 44.7% [12,13,14,16,18,19,20]. Our result is in line with those of Meisner et al. [16], who reported a seroprevalence of 40.7% in the USA, and Panei et al. [20], who reported a seroprevalence of 44.7% in Argentina. Even in Africa, where pets generally maintain an outdoor lifestyle with more limited contact with their owners, our findings support the notion of frequent human-to-pet transmission of SARS-CoV-2.

Our study detected SARS-CoV-2 RNA in 14% of pets, including 9 of 86 (10.5%) dogs and 5 of 14 (35.7%) cats. This rate is high compared to those of Meisner et al. in the USA (5.3%) and Jairak et al. in Thailand (9.1%) [16,17], but lower than that reported by Calvet (33.3%) in Brazil [15]. The difference in prevalence observed between these studies could be explained by several factors, including the number of animals tested and the duration between the onset of the owner’s symptoms and the time the animal was sampled.

Our seroprevalence data suggest that infection rates are likely higher than those observed using RT-qPCR, as SARS-CoV-2 RNA had not been detected in 25.7% (18/70) of the serologically positive pets later tested by MIA. This difference in detection between MIA and RT-qPCR may be due to a smaller detection window in RT-PCR than in MIA.

Pet samples that remained positive for SARS-CoV-2 RNA following an initial positive test included those collected with nasopharyngeal swabs 1 to 12 days and 1 to 17 days in dogs and cats, respectively, as well as rectal swabs collected 1 to 3 days later in dogs. Variable viral shedding periods ranging from one to 31 days have been reported in naturally infected dogs and cats [15,17,28,34]. These results highlight a potentially lengthy period of SARS-CoV-2 viral shedding in pets, which may increase the risk of contamination of humans or wildlife via close contact or via contaminated biological material such as respiratory drops, saliva, or even feces.

Moreover, 37.9% (12/32) of SARS-CoV-2-positive pets presented mild clinical signs of infection. Interestingly, we observed that the nature of the clinical signs of infection (respiratory vs. gastrointestinal) was related to the presence of RNA collected on nasopharyngeal and rectal swabs, respectively.

Phylogenetic analysis of the ten viral genomes collected from dogs and cats showed them clustering with sequences in clades 20A, 20B, and 20H. Among the four sequences belonging to clade 20A, the two cat sequences detected in February and October 2021 correspond to variant B.1.640.1 sequences detected in humans in RoC between January and November 2021 [35]. Unfortunately, the absence of viral sequences from owners means that we cannot definitively demonstrate that pet infections were acquired from their COVID-19 + owners. Still, this route of infection certainly seems the most parsimonious explanation. Furthermore, the observation of the high circulation of 20A variants in the human population of Brazzaville between January and November 2022 strongly suggests that the transmission was from human to animal.

A total of 5 SARS-CoV-2 sequences collected between May and August 2021 belong to the 20H clade. Surprisingly, no sequences belonging to clade 20H were found among the SARS-CoV-2 sequences available in GISAID collected from humans in RoC. However, virus from the 20H clade was first detected in South Africa [36] and had been detected circulating in countries neighboring RoC, such as the Democratic Republic of the Congo (DRC), Gabon, the Central African Republic, Cameroon, and Angola between December 2020 and March 2022 [37]. Given the intercommunication and circulation of people in this region, it is very likely that this variant was also circulating in the RoC during this period but was not detected due to the lack of routine sequencing of COVID-19+ human samples in the RoC and the lack of a high-capacity sequencing platform for SARS-CoV-2.

Interestingly, the viral sequence from Cat-103 sampled in October 2021 exhibited an anomalous profile. This virus clustered within clade 20B according to Nextclade analyses, whereas phylogenetic analyses found it to cluster within the 20H clade. Similar to the 20H virus clade, viruses of the 20B clade had also not been found among the Congolese human SARS-CoV-2 sequences. Nevertheless, 20B sequences were circulating in RoC’s neighboring countries, such as DRC and Cameroon, between May 2020 and January 2022 (https://www.gisaid.org/ accessed on 4 February 2022). This ambiguity could most likely be a result of recombination between the viruses of clades 20B and 20H. Indeed, the occurrence of recombination in SARS-CoV-2 sequences has been described in several studies [38,39,40,41,42]. Nevertheless, this ambiguity could also be the result of the accumulation of independent mutations during viral replication due to the large RNA genome of coronaviruses and the low fidelity of their RNA-dependent RNA polymerase [43]. It is unclear and impossible to determine in our study whether this anomalous variant was present in the owner before transmission to the cat or could be generated directly in the cat due to a break in the human chain of transmission.

The high incidence of SARS-CoV-2 infection in cats and dogs in RoC could indicate a significant and understudied risk of SARS-CoV-2 transmission and spread to other susceptible animals. Transmission to wildlife has already been observed in white-tailed deer in the USA, with this species possibly becoming a reservoir host for SARS-CoV-2 [44]. In addition, the transmission of SARS-CoV-2 to a new host species may drive viral evolution as the population adapts to the new host environment and immune pressure. In the case of possible subsequent wildlife-to-human transmission, such viral evolution may cause unknown and unpredictable changes in pathogenicity and transmission dynamics in humans. A spillback from white-tailed deer to humans has been suggested in Canada from a virus carrying numerous mutations characteristic of SARS-CoV-2 strains infecting white-tailed deer [45]. Our study has focused on the capital of RoC, Brazzaville, which is the area reporting the most cases of infection but also the one that has been tested the most during the epidemic. More studies must be performed to better understand the threat that SARS-CoV-2 represents to pets and its possible transmission to RoC wildlife, especially in remote villages in the tropical rainforest of the Congo Basin.

Domestic animals living in RoC, in particular, and in Africa, in general, have an indoor/outdoor lifestyle with frequent interactions with the rich biodiversity of the surrounding wildlife [46]. Our results highlight the importance of implementing a One-Health approach that includes pets and wild animals in SARS-CoV-2 surveillance in low- and medium-income countries, perhaps even more so in regions with such high local biodiversity as that found in the countries of the Congo Basin.

## Figures and Tables

**Table 1:** Features of pets testing positive for SARS-CoV-2 by RT-qPCR and/or MIA in RoC.

Animal_ID	Age (Year)	Sex	Breeds	Clinical Signs	Days from Owner’s OS	Date of Owner’s COVID-19+	Date of 1st Animal’s Sampling	RT-qPCR	MIA
Cat-020	1.5	F	European shorthair	-	As	19/02/2021	23/02/2021	35.22	8926/2874
Cat-021	0.6	M	European shorthair	-	As	19/02/2021	23/02/2021	0.0	14,017/5795
Cat-022	0.6	F	European shorthair	-	As	19/02/2021	24/02/2021	0.0	1782/479
Cat-030	0.3	F	Local	-	16	12/03/2021	15/03/2021	33.47	5467.5/2420
Cat-069	0.25	F	Local	S	17	31/05/2021	07/06/2021	32.20/30.20	7903/3282
Cat-103	1	M	Local	F, LA,LW	13	23/10/2021	28/10/2021	27.08/25.82/27.24	2820/1589
Cat-104	1.4	M	Local	-	9	24/10/2021	28/10/2021	24.44/22.69/23.94	2447/1384.5
Dog-002	8	M	German Shepherd	F, LW	24	02/02/2021	05/02/2021	0.0	4527/1596.5
Dog-004	2	F	German Shepherd	F, LW	24	02/02/2021	05/02/2021	0.0	5374.5/2122
Dog-006	3	F	Poodle	-	8	06/02/2021	08/02/2021	0.0	6809/2521
Dog-008	0.83	M	Poodle	-	8	06/02/2021	08/02/2021	0.0	1554/691
Dog-017	7	M	German Shepherd	F, OW	As	17/02/2021	19/02/2021	0.0	1285/643.5
Dog-019	0.75	M	Pitbull	-	As	17/02/2021	22/02/2021	0.0	2469.5/1217.5
Dog-028	4.4	M	Malinois Shepherd	-	9	08/03/2021	10/03/2021	0.0	6273.5/2505
Dog-031	7	M	Poodle	-	16	12/03/2021	18/03/2021	0.0	12,415/5369
Dog-032	2	M	Local	-	13	12/03/2021	15/03/2021	34.47/30.02	6420/2118
Dog-048	3	M	Crossbreed	-	As	06/04/2021	12/04/2021	0.0	2984/1034
Dog-051	13	F	Poodle	F, D, AA	13	10/04/2021	16/04/2021	0.0	1935.5/492.5
Dog-052	6	M	Malinois Shepherd	Fe, F, D, LW	As	16/04/2021	19/04/2021	27.90	4666/1247.5
Dog-055	0.58	M	German Shepherd	-	18	18/04/2021	22/04/2021	0.0	1604/400
Dog-062	3	M	Crossbreed	-	16	21/05/2021	25/05/2021	34.40	NA
Dog-065	3	F	Crossbreed	-	As	28/05/2021	31/05/2021	0.0	1846/435
Dog-071	2	M	Poodle	-	As	10/06/2021	11/06/2021	0.0	2712/774.5
Dog-072	2	M	Crossbreed	-	As	08/06/2021	14/06/2021	0.0	1778/481
Dog-074	2	M	German Shepherd	-	As	14/06/2021	17/06/2021	0.0	1878.5/181
Dog-075	3	M	Poodle	-	As	14/06/2021	21/06/2021	0.0	4488/1168
Dog-078	9	M	Siberian husky	-	17	22/06/2021	25/06/2021	35.40	3792.5/1230
Dog-082	0.3	F	Poodle	S	7	09/07/2021	13/07/2021	27.80/27.20/28.34	10,334/4416
Dog-083	0.3	M	Crossbreed	-	As	14/07/2021	16/07/2021	33.15	111/58
Dog-085	5	M	Crossbreed	G	As	09/07/2021	19/07/2021	31.66/35.90	382.5/58
Dog-087	2	F	Local	LA,LW	12	29/07/2021	03/08/2021	34.25/36.08	5207/1862.5
Dog-094	9	M	Poodle	F	17	15/08/2021	26/08/2021	29.42/28.24/27.30	3842/628.5

With: As (asymptomatic), -(none), F (fever), F (fatigue) LA (loss of appetite), LW (loss of weight), D (diarrhea), G (gastroenteric), S (sneezing), OW (overweight), OS (onset of symptoms), and NA (not applicable). The MIA cut-off values of cats were 817 for RBD and 210 for Stri. For dogs, it was 1174 for RBD and 349 for Stri.

**Table 2:** Rate of SARS-CoV-2 detection by RT-qPCR and MIA in pets from Congo.

Species	RT-qPCR	MIA
	N (%)	N (%)
Cats	5/14 (35.7)	7/10 (70)
Dogs	9/86 (10.5)	22/65 (33.8)
Total (%)	14/100 (14.0)	29/75 (38.6)

## Data Availability

The authors confirm that all data supporting the findings of this study are available in this article and its supplementary materials.

## Supplementary Materials

The following supporting information can be downloaded at: https://www.mdpi.com/article/10.3390/v15040933/s1, Figure S1: Pets’ qRT-PCR positive profiles; Table S1: Pets’ features and results; Table S2: Pets’ Luminex data.
