# Supplementary material for: Whole-Genome Characterization of SARS-CoV-2 Reveals Simultaneous Circulation of Three Variants and a Putative Recombination (20B/20H) in Pets, Brazzaville, Republic of the Congo
Source: Viruses. 2023 Apr 9;15(4):933. doi: 10.3390/v15040933 (PMC10142050; doi:10.3390/v15040933)
Supplement: Supplementary file 1 [file viruses-15-00933-s001.zip › viruses-2251214-supplementary.pdf]

Table S1-Pets' features and results

Animal_ID	Breeds	Districs	Sex	Age (year)	Clinical signs	Dates of swab sampling	RT-qPCR (CT) Nasopharyngeal swab ORF1ab	N	S	Rectal swab ORF1ab	N	S	Dates of serum sampling	MIA RBD	S
**Dog-001**	Crossbreed	MFILOU	F	5	Fatigue, overweight	05/02/2021	ND	NA	NA	ND	NA	NA	09/08/2021	87	42
						08/02/2021	ND	NA	NA	ND	NA	NA			
**Dog-002**	German Shepherd	MOUNGALI	M	8	Fatigue, weight loss	05/02/2021	ND	NA	NA	ND	NA	NA	06/12/2021	4527	159,5
						08/02/2021	ND	NA	NA	ND	NA	NA			
**Dog-003**	German Shepherd	MOUNGALI	F	4	Fatigue, weight loss	05/02/2021	ND	NA	NA	ND	NA	NA	NA	NA	NA
						08/02/2021	ND	NA	NA	ND	NA	NA			
**Dog-004**	German Shepherd	MOUNGALI	F	2	Fatigue, weight loss	05/02/2021	ND	NA	NA	ND	NA	NA	09/08/2021	5374.5	2122
						09/02/2021	ND	NA	NA	ND	NA	NA			
**Dog-005**	Local	MOUNGALI	M	1.5	None	08/02/2021	ND	NA	NA	ND	NA	NA	NA	NA	NA
						10/02/2021	ND	NA	NA	ND	NA	NA			
**Dog-006**	Poodle	DJIRI	F	3	None	08/02/2021	ND	NA	NA	ND	NA	NA	09/08/2021	6809	2521
						10/02/2021	ND	NA	NA	ND	NA	NA			
**Dog-007**	Crossbreed	DJIRI	M	0.6	None	08/02/2021	ND	NA	NA	ND	NA	NA	09/08/2021	598	109
						10/02/2021	ND	NA	NA	ND	NA	NA			
**Dog-008**	Poodle	DJIRI	M	0.8	None	08/02/2021	ND	NA	NA	ND	NA	NA	10/08/2021	1554	691
						11/02/2021	ND	NA	NA	ND	NA	NA			
**Dog-009**	Chihuahua	OUENZE	F	1.5	None	10/02/2021	ND	NA	NA	ND	NA	NA	NA	NA	NA
						12/02/2021	ND	NA	NA	ND	NA	NA			
**Dog-010**	Crossbreed	MADIBOU	M	5	Fatigue	11/02/2021	ND	NA	NA	ND	NA	NA	12/08/2021	272.5	78
						15/02/2021	ND	NA	NA	ND	NA	NA			
**Dog-011**	Crossbreed	MADIBOU	M	5	Fatigue	11/02/2021	ND	NA	NA	ND	NA	NA	12/08/2021	332	121
						15/02/2021	ND	NA	NA	ND	NA	NA			
**Dog-012**	Local	MADIBOU	F	3	Fatigue	11/02/2021	ND	NA	NA	ND	NA	NA	12/08/2021	195	70
						15/02/2021	ND	NA	NA	ND	NA	NA			

**Dog-013**	Pitbull	DJIRI	F	0.3	None	19/02/2021	ND	NA	NA	ND	NA	NA	NA	NA	NA
						22/02/2021	ND	NA	NA	ND	NA	NA			
**Dog-014**	Boerbull	DJIRI	M	0.3	None	19/02/2021	ND	NA	NA	ND	NA	NA	10/08/2021	185	80
						22/02/2021	ND	NA	NA	ND	NA	NA			
**Dog-015**	Pitbull	DJIRI	M	0.2	None	19/02/2021	ND	NA	NA	ND	NA	NA	NA	NA	NA
						22/02/2021	ND	NA	NA	ND	NA	NA			
**Dog-016**	Boerbull	DJIRI	F	0.2	None	19/02/2021	ND	NA	NA	ND	NA	NA	10/08/2021	125	63
						22/02/2021	ND	NA	NA	ND	NA	NA			
**Dog-017**	German Shepherd	DJIRI	M	7	Fatigue , overweight	19/02/2021	ND	NA	NA	ND	NA	NA	09/08/2021	1285	643.5
						22/02/2021	ND	NA	NA	ND	NA	NA			
**Dog-018**	Local	DJIRI	M	7	None	22/02/2021	ND	NA	NA	ND	NA	NA	10/08/2021	444	43.5
						24/02/2021	ND	NA	NA	ND	NA	NA			
**Dog-019**	Pitbull	DJIRI	M	0.7	None	22/02/2021	ND	NA	NA	ND	NA	NA	10/08/2021	2469.5	1217.5
						24/02/2021	ND	NA	NA	ND	NA	NA			
**Cat-020**	European shorthair	MOUNGALI	F	1. 5	None	23/02/2021	ND	NA	35.22	ND	NA	NA	02/09/2021	8926	2874
**Cat-021**	European shorthair	MOUNGALI	M	0.6	None	23/02/2021	ND	NA	NA	ND	NA	NA	02/09/2021	14017	5795
**Cat-022**	European shorthair	MOUNGALI	F	0.6	None	24/02/2021	ND	NA	NA	ND	NA	NA	08/09/2021	1782	479
**Dog-023**	Poodle	MAKELEKELE	F	3	None	03/03/2021	ND	NA	NA	ND	NA	NA	NA	NA	NA
						05/03/2021	ND	NA	NA	ND	NA	NA			
**Cat-024**	Local	MAKELEKELE	F	3	None	03/03/2021	ND	NA	NA	ND	NA	NA	NA	NA	NA
						05/03/2021	ND	NA	NA	ND	NA	NA			
**Cat-025**	Local	MAKELEKELE	F	3	None	03/03/2021	ND	NA	NA	ND	NA	NA	NA	NA	NA
						05/03/2021	ND	NA	NA	ND	NA	NA			
**Cat-027**	German Shepherd	MOUNGALI	M	4	None	03/03/2021	ND	NA	NA	ND	NA	NA	NA	NA	NA
**Dog-026**	Local	MAKELEKELE	M	3	None	05/03/2021	ND	NA	NA	ND	NA	NA	04/09/2021	147	41
**Dog-028**	Malinois Shepherd	MFILOU	M	4.4	None	10/03/2021	ND	NA	NA	ND	NA	NA	13/08/2021	6273.5	2505
						12/03/2021	ND	NA	NA	ND	NA	NA			
**Dog-029**	Crossbreed	MADIBOU	M	6	None	10/03/2021	ND	NA	NA	ND	NA	NA	11/08/2021	511	260
						12/03/2021	ND	NA	NA	ND	NA	NA			
**Cat-030**	Local	DJIRI	F	0.3	None	15/03/2021	ND	ND	33.47	ND	ND	ND	17/08/2021	5467.5	2420

**Dog-031**	Poodle	DJIRI	M	7	None	15/03/2021	ND	ND	ND	ND	ND	ND	13/08/2021	12415	5369
**Dog-032**	Local	MADIBOU	M	2	None	15/03/2021	ND	ND	ND	ND	34.47	30.02	25/08/2021	6420	2118
						17/03/2021	ND	ND	ND	ND	35.8	ND			
						19/03/2021	ND	ND	ND	ND	ND	ND			
**Dog-033**	Shelpherd	POTO-POTO	F	1.6	None	16/03/2021	ND	ND	ND	ND	ND	ND	19/08/2021	93	48.5
						18/03/2021	ND	ND	ND	ND	ND	ND			
**Cat-034**	Local	MAKELEKELE	M	1	None	18/03/2021	ND	ND	ND	ND	ND	ND	21/08/2021	147	115
						22/03/2021	ND	ND	ND	ND	ND	ND			
**Dog-035**	Shelpherd	POTO-POTO	M	0.3	None	23/03/2021	ND	ND	ND	ND	ND	ND	NA	NA	NA
						25/03/2021	ND	ND	ND	ND	ND	ND			
**Dog-036**	Shelpherd	POOL	M	0.5	None	26/03/2021	ND	ND	ND	ND	ND	ND	24/08/2021	118	58
						29/03/2021	ND	ND	ND	ND	ND	ND			
**Dog-037**	Poodle	POTO-POTO	F	0.8	Fever, fatigue, gastroenteritis, loss of appetite	29/03/2021	ND	ND	ND	ND	ND	ND	17/09/2021	255	77
						31/03/2021	ND	ND	ND	ND	ND	ND			
**Dog-038**	Shelpherd	TALANGAÏ	F	0.3	None	29/03/2021	ND	ND	ND	ND	ND	ND	NA	NA	NA
						31/03/2021	ND	ND	ND	ND	ND	ND			
**Dog-039**	Malinois Shepherd	TALANGAÏ	F	2	None	29/03/2021	ND	ND	ND	ND	ND	ND	NA	NA	NA
						31/03/2021	ND	ND	ND	ND	ND	ND			
**Dog-040**	Boerbull	TALANGAÏ	F	0.4	None	29/03/2021	ND	ND	ND	ND	ND	ND	NA	NA	NA
						31/03/2021	ND	ND	ND	ND	ND	ND			
**Dog-041**	Poodle	TALANGAÏ	F	5	Fever, weight loss	29/03/2021	ND	ND	ND	ND	ND	ND	NA	NA	NA
						31/03/2021	ND	ND	ND	ND	ND	ND			
**Dog-042**	Malinois Shepherd	TALANGAÏ	F	0.6	Fatigue, Weight loss, breathing difficulty	29/03/2021	ND	ND	ND	ND	ND	ND	NA	NA	NA
**Dog-043**	Crossbreed	MFILOU	M	2.5	None	02/04/2021	ND	ND	ND	ND	ND	ND	NA	NA	NA
						05/04/2021	ND	ND	ND	ND	ND	ND			
**Dog-044**	Local	MFILOU	F	5	None	02/04/2021	ND	ND	ND	ND	ND	ND	25/08/2021	247	77.5
						05/04/2021	ND	ND	ND	ND	ND	ND			
**Dog-045**	German Shepherd	MAKELEKELE	M	4	None	02/04/2021	ND	ND	ND	ND	ND	ND	21/08/2021	163	58
						05/04/2021	ND	ND	ND	ND	ND	ND			

**Dog-046**	German Shepherd	MAKELEKELE	M	4	None	02/04/2021	ND	ND	ND	ND	ND	ND	21/08/2021	720	114
						05/04/2021	ND	ND	ND	ND	ND	ND			
**Dog-047**	Crossbreed	POTO-POTO	F	10	None	12/04/2021	ND	ND	ND	ND	ND	ND	19/08/2021	198	46.5
						14/04/2021	ND	ND	ND	ND	ND	ND			
**Dog-048**	Crossbreed	POTO-POTO	M	3	None	12/04/2021	ND	ND	ND	ND	ND	ND	19/082021	2984	1034
						14/04/2021	ND	ND	ND	ND	ND	ND			
**Dog-049**	German Shepherd	MADIBOU	M	5	None	13/04/2021	ND	ND	ND	ND	ND	ND	25/08/2021	455	101.5
						15/04/2021	ND	ND	ND	ND	ND	ND			
**Dog-050**	Poodle	MOUNGALI	F	13	None	16/04/2021	ND	ND	ND	ND	ND	ND	17/09/2021	455	101.5
						18/04/2021	ND	ND	ND	ND	ND	ND			
**Dog-051**	Poodle	MOUNGALI	F	13	Fatigue, diarrhean, anal swelling	16/04/2021	ND	ND	ND	ND	ND	ND	27/09/2021	684	361
						18/04/2021	ND	ND	ND	ND	ND	ND			
**Dog-052**	Berger malinois	OUENZE	M	6	Fever, Fever , weight loss, diarhea , eczema	19/04/2021	ND	ND	ND	ND	ND	27.90	28/08/2021	1935.5	492.5
						21/04/2021	ND	ND	ND	ND	ND	34.80	NA	NA	NA
						23/04/2021	ND	ND	ND	ND	ND	ND			
**Dog-053**	Poodle	MOUNGALI	M	0.2	None	19/04/2021	ND	ND	ND	ND	ND	ND	NA	NA	NA
**Dog-054**	Crossbreed	OUENZE	M	7	Eczema	21/04/2021	ND	ND	ND	ND	ND	ND	28/08/2021	408.5	325
**Dog-055**	Shelpherd	MOUNGALI	M	0.6	None	22/04/2021	ND	ND	ND	ND	ND	ND	25/08/2021	1604	400
						27/04/2021	ND	ND	ND	ND	ND	ND			
**Dog-056**	Shelpherd	MAKELEKELE	F	1.7	None	27/04/2021	ND	ND	ND	ND	ND	ND	NA	NA	NA
						29/04/2021	ND	ND	ND	ND	ND	ND			
**Dog-057**	German Shepherd	BACONGO	M	5	None	06/05/2021	ND	ND	ND	ND	ND	ND	21/08/2021	79	36
						10/05/2021	ND	ND	ND	ND	ND	ND			
**Dog-058**	German Shepherd	BACONGO	F	5	None	06/05/2021	ND	ND	ND	ND	ND	ND	21/08/2021	92	38.5
						10/05/2021	ND	ND	ND	ND	ND	ND			
**Cat-059**	Local	MFILOU	F	0.2	None	06/05/2021	ND	ND	ND	ND	ND	ND	23/08/2021	36	20
**Cat-060**	Local	MFILOU	F	0.6	None	21/05/2021	ND	ND	ND	ND	ND	ND	NA	NA	NA
						24/05/2021	ND	ND	ND	ND	ND	ND			
**Dog-061**	Local	MFILOU	M	4	None	21/05/2021	ND	ND	ND	ND	ND	ND	23/08/2021	396	57
						24/05/2021	ND	ND	ND	ND	ND	ND			
**Dog-062**	Crossbreed	MOUNGALI	M	3	None	25/05/2021	ND	34.40	ND	ND	ND	ND	NA	NA	NA
						27/05/2021	ND	ND	ND	ND	ND	ND			

**Dog-063**	Crossbreed	MOUNGALI	F	8	None	25/05/2021	ND	ND	ND	ND	ND	ND	NA	NA	NA
						27/05/2021	ND	ND	ND	ND	ND	ND			
**Dog-064**	Local	POOL	F	2	None	31/05/2021	ND	ND	ND	ND	ND	ND	25/08/2021	141	96
						02/06/2021	ND	ND	ND	ND	ND	ND			
**Dog-065**	Crossbreed	POOL	F	3	None	31/05/2021	ND	ND	ND	ND	ND	ND	25/08/2021	1846	435
						02/06/2021	ND	ND	ND	ND	ND	ND			
**Dog-066**	Crossbreed	POOL	F	2	None	31/05/2021	ND	ND	ND	ND	ND	ND	25/08/2021	295	80.5
						02/06/2021	ND	ND	ND	ND	ND	ND			
**Dog-067**	Local	MOUNGALI	M	7	None	02/06/2021	ND	ND	ND	ND	ND	ND	NA	NA	NA
**Dog-068**	Poodle	MOUNGALI	F	0.5	None	04/06/2021	ND	ND	ND	ND	ND	ND	31/08/2021	215	86.5
						07/06/2021	ND	ND	ND	ND	ND	ND			
**Cat-069**	Local	DJIRI	F	0.2	Sneezing	07/06/2021	32.20	30.20	ND	ND	ND	ND	08/09/2021	7903	3282
						09/06/2021	32.50	34.10	ND	ND	ND	ND			
						11/06/2021	ND	36.80	ND	ND	ND	ND			
						14/06/2021	ND	ND	ND	ND	ND	ND			
**Dog-070**	Poodle	BACONGO	M	0.3	None	11/06/2021	ND	ND	ND	ND	ND	ND	NA	NA	NA
						14/06/2021	ND	ND	ND	ND	ND	ND			
**Dog-071**	Poodle	BACONGO	M	2	None	11/06/2021	ND	ND	ND	ND	ND	ND	31/08/2021	2712	774.5
						14/06/2021	ND	ND	ND	ND	ND	ND			
**Dog-072**	Crossbreed	MOUNGALI	M	2	None	14/06/2021	ND	ND	ND	ND	ND	ND	31/08/2021	1778	481
						17/06/2021	ND	ND	ND	ND	ND	ND			
**Dog-073**	Crossbreed	MOUNGALI	M	0.5	None	17/06/2021	ND	ND	ND	ND	ND	ND	04/09/2021	80	64
						21/06/2021	ND	ND	ND	ND	ND	ND			
**Dog-074**	German Shepherd	MOUNGALI	M	2	None	17/06/2021	ND	ND	ND	ND	ND	ND	17/09/2021	1878.5	181
						22/06/2021	ND	ND	ND	ND	ND	ND			
**Dog-075**	Poodle	MOUNGALI	M	3	None	21/06/2021	ND	ND	ND	ND	ND	ND	01/09/2021	4488	1168
						23/06/2021	ND	ND	ND	ND	ND	ND			
**Dog-076**	Local	MAKELEKELE	F	11	None	25/06/2021	ND	ND	ND	ND	ND	ND	08/09/2021	96	29
**Dog-077**	Crossbreed	MOUNGALI	F	9	None	25/06/2021	ND	ND	ND	ND	ND	ND	02/09/2021	114	33
						28/06/2021	ND	ND	ND	ND	ND	ND			
**Dog-078**	Siberian Husky	MOUNGALI	M	9	None	25/06/2021	ND	35.40	ND	ND	ND	ND	02/09/2021	3792.5	1230
						28/06/2021	ND	ND	ND	ND	ND	ND			

**Dog-079**	Crossbreed	DJIRI	M	1.7	None	25/06/2021	ND	ND	ND	ND	ND	ND	01/09/2021	94.5	142
						28/06/2021	ND	ND	ND	ND	ND	ND			
**Dog-080**	Crossbreed	MOUNGALI	M	0.7	None	05/07/2021	ND	ND	ND	ND	ND	ND	01/09/2021	132	65
						07/07/2021	ND	ND	ND	ND	ND	ND			
**Dog-081**	Crossbreed	MOUNGALI	M	0.6	None	12/07/2021	ND	ND	ND	ND	ND	ND	10/09/2021	83	107
						14/07/2021	ND	ND	ND	ND	ND	ND			
**Dog-082**	Poodle	MOUNGALI	F	0.3	Sneezing	13/07/2021	27.80	27.20	28.34	ND	ND	ND	19/09/2021	10334	4416
						16/07/2021	30.75	28.68	28.26	ND	ND	ND			
						21/07/2021	34.00	32.50	31.44	ND	ND	ND			
						24/07/2021	ND	36.80	ND	ND	ND	ND			
						28/07/2021	ND	ND	ND	ND	ND	ND			
**Dog-083**	Crossbreed	OUENZE	M	0.3	None	16/07/2021	ND	ND	ND	ND	ND	ND	02/09/2021	111	58
						19/07/2021	ND	ND	ND	ND	33.15	ND			
						21/07/2021	ND	ND	ND	ND	ND	ND			
**Dog-084**	Malinois Shepherd	POOL	M	1.5	None	16/07/2021	ND	ND	ND	ND	ND	ND	NA	NA	NA
						19/07/2021	ND	ND	ND	ND	ND	ND			
**Dog-085**	Crossbreed	BACONGO	M	5	Gastroenteric	19/07/2021	ND	ND	ND	ND	31.66	35.90	02/09/2021	382.5	58
						21/07/2021	ND	ND	ND	ND	33.99	36.77			
						23/07/2021	ND	ND	ND	ND	ND	ND			
**Dog-087**	Local	MADIBOU	F	2	Weight loss , loss of appetite	03/08/2021	ND	ND	ND	ND	34.25	36.08	02/09/2021	5207	1862.5
						05/08/2021	ND	ND	ND	ND	ND	ND			
**Dog-092**	Labrador	MOUNGALI	M	0.2	Fatigue, cayor worms , sores	04/08/2021	ND	ND	ND	ND	ND	ND	15/09/2021	224.5	55
						06/08/2021	ND	ND	ND	ND	ND	ND			
**Dog-093**	Poodle	MOUNGALI	M	2	depression , mood swings	04/08/2021	ND	ND	ND	ND	ND	ND	15/09/2021	299	96
						06/08/2021	ND	ND	ND	ND	ND	ND			
**Dog-094**	Poodle	OUENZE	M	9	Fatigue	26/08/2021	29.42	28.24	27.30	ND	ND	ND	19/10/2021	3842	628.5
						30/08/2021	33.74	29.18	29.90	ND	ND	ND			
						01/09/2021	ND	34.49	ND	ND	ND	ND			
						04/09/2021	ND	ND	ND	ND	ND	ND			
**Dog-095**	Poodle	MAKELEKELE	F	5	Fatigue	19/10/2021	ND	ND	NA	ND	ND	NA	06/12/2021	44	36.5
**Dog-096**	Poodle	DJIRI	M	2	None	20/10/2021	ND	ND	NA	ND	ND	NA	06/12/2021	47	39
						23/10/2021	ND	ND	NA	ND	ND	NA			

**Dog-097**	Crossbreed	POTO-POTO	F	0.2	None	21/10/2021	ND	ND	NA	ND	ND	NA	06/12/2021	47	42
						25/10/2021	ND	ND	NA	ND	ND	NA			
**Dog-098**	Poodle	MOUNGALI	M	5	Limping, fractured right front leg	26/10/2021	ND	ND	NA	ND	ND	NA	06/12/2021	51	43
						30/10/2021	ND	ND	NA	ND	ND	NA			
**Cat-099**	Local	MOUNGALI	M	2	None	27/10/2021	ND	ND	NA	ND	ND	NA	06/12/2021	52.5	44
						30/10/2021	ND	ND	NA	ND	ND	NA			
**Dog-100**	Crossbreed	MADIBOU	M	2	None	28/10/2021	ND	ND	NA	ND	ND	NA	06/12/2021	49	42
						30/10/2021	ND	ND	NA	ND	ND	NA			
**Dog-101**	Crossbreed	MADIBOU	F	7	None	28/10/2021	ND	ND	NA	ND	ND	NA	06/12/2021	45	37.5
						30/10/2021	ND	ND	NA	ND	ND	NA			
**Dog-102**	Crossbreed	MADIBOU	M	0.5	None	28/10/2021	ND	ND	NA	ND	ND	NA	06/12/2021	44	38
						30/10/2021	ND	ND	NA	ND	ND	NA			
**Cat-103**	Local	MADIBOU	M	1	Fatigue , weight loss	28/10/2021	27.08	25.82	27.24	ND	ND	NA	06/12/2021	2820	1589
						02/11/2021	30.14	28.33	26.00	ND	ND	NA			
						04/11/2021	ND	34.46	36.18	ND	ND	NA			
						08/11/2021	ND	ND	ND	ND	ND	NA			
**Cat-104**	Local	MFILOU	M	1.3	None	28/10/2021	24.44	22.69	23.94	ND	ND	NA	06/12/2021	2447	1384.5
						02/11/2021	28.23	25.80	27.08	ND	ND	NA			
						04/11/2021	27.74	28.00	29.11	ND	ND	NA			
						13/11/2021	34.22	34.26	32.96	ND	ND	NA			
						20/11/2021	ND	ND	NA	ND	ND	NA			
**Dog-105**	Crossbreed	DJIRI	M	3	None	04/11/2021	ND	ND	NA	ND	ND	NA	06/12/2022	NA	NA

With ND, not detected; NA, not applicable

Figure S1-Pets's qRT-PCR positive profiles

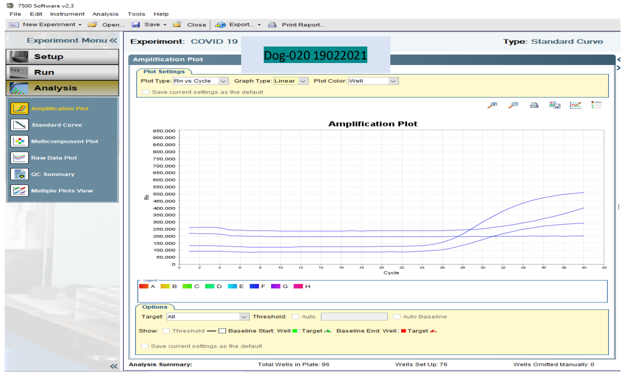

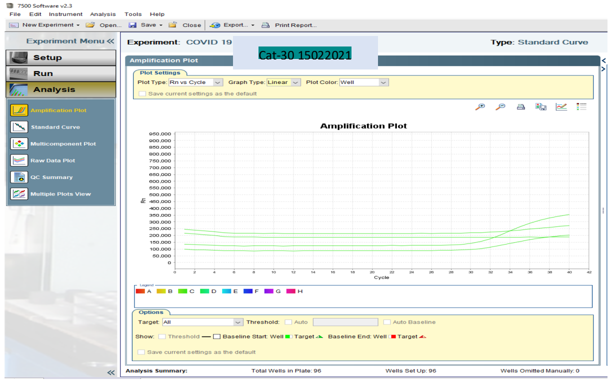

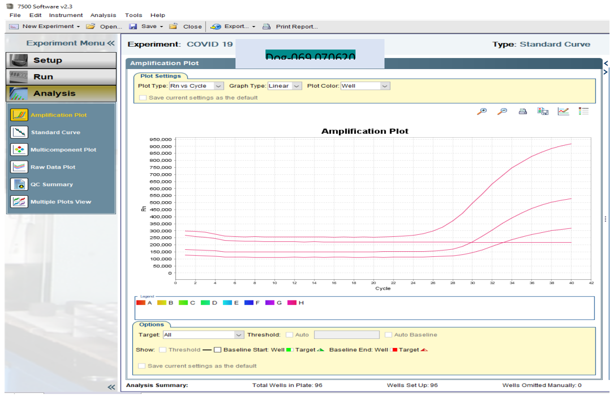

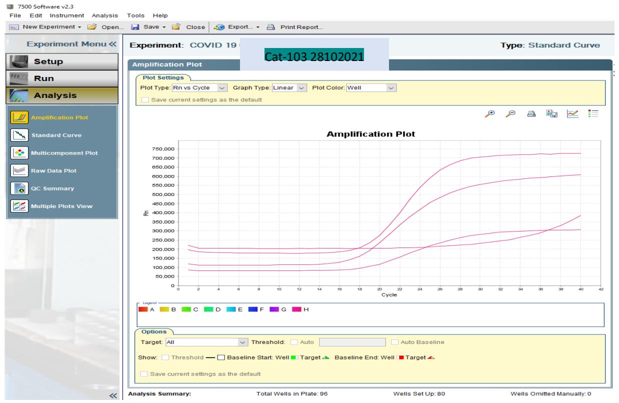

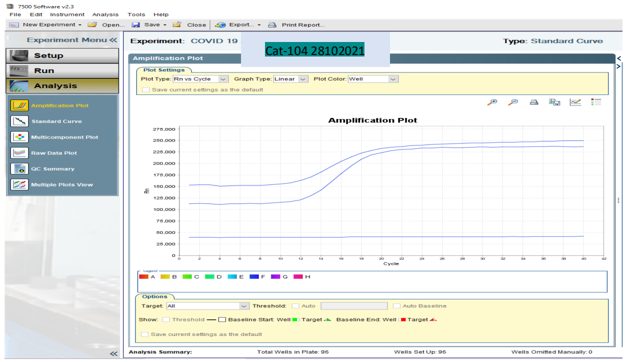

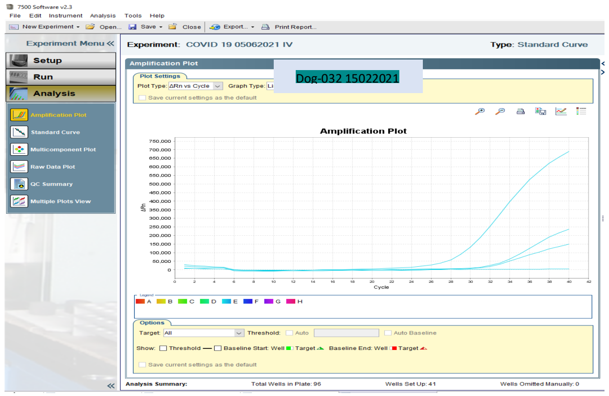
Dog-032 15022021
Experiment: COVID 19 05062021 IV
Type: Standard Curve
Amplification Plot

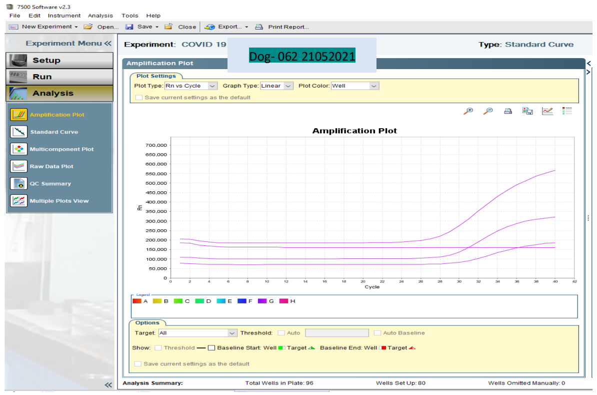
Dog- 062 21052021
Experiment: COVID 19
Type: Standard Curve
Amplification Plot

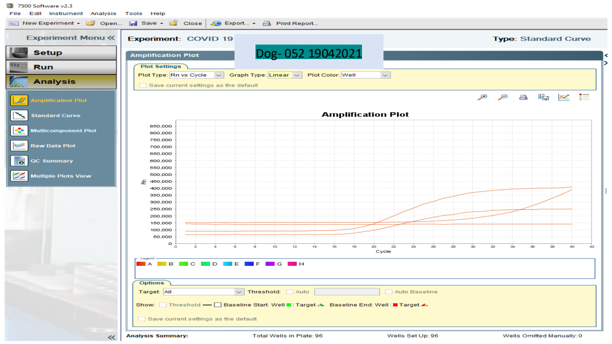
Dog- 052 19042021
Experiment: COVID 19
Type: Standard Curve
Amplification Plot

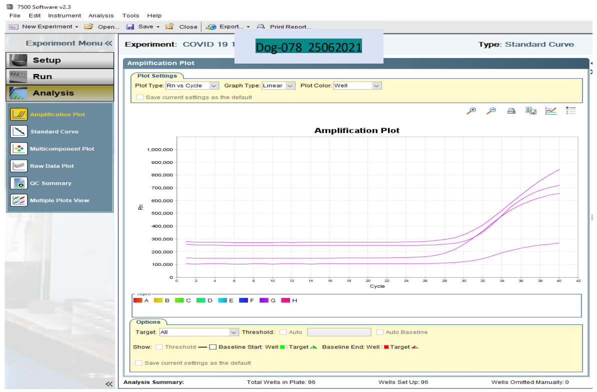
Dog-078 25062021
Experiment: COVID 19
Type: Standard Curve
Amplification Plot

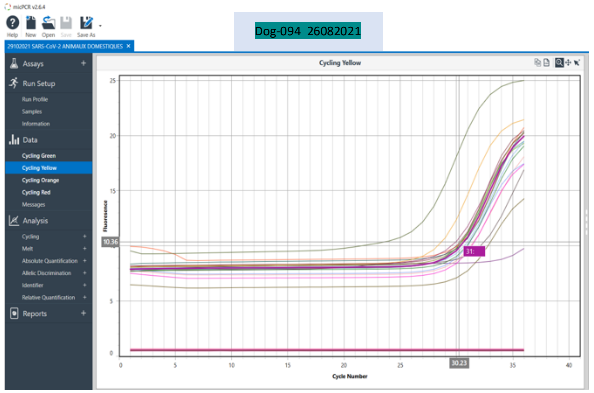
Dog-094 26082021
Cycling Yellow
Cycle Number

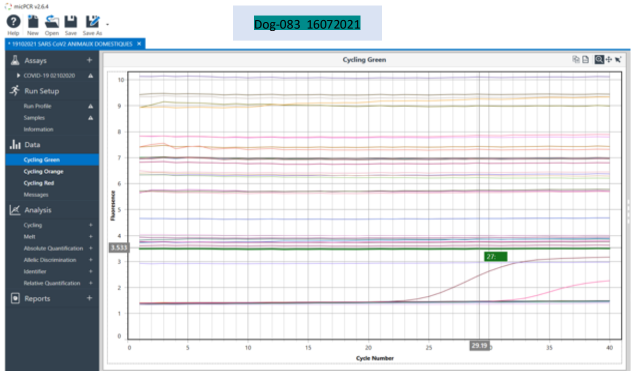
Dog-083 16072021
Cycling Green
Cycle Number

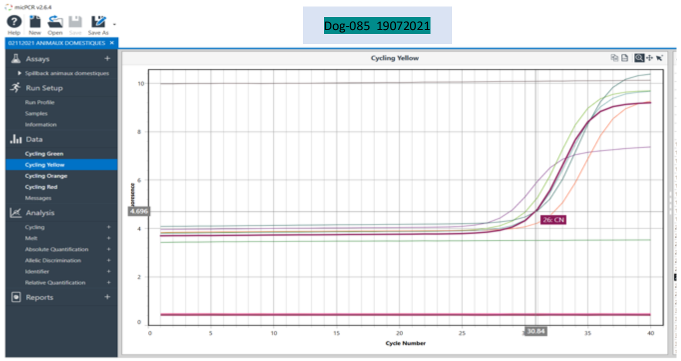
Dog-085 19072021
Cycling Yellow
Cycle Number

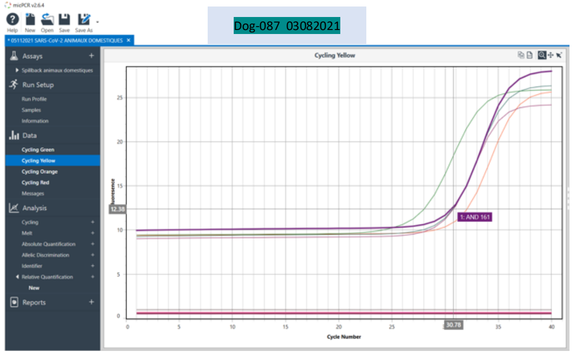
Dog-087 03082021
Cycling Yellow
Cycle Number

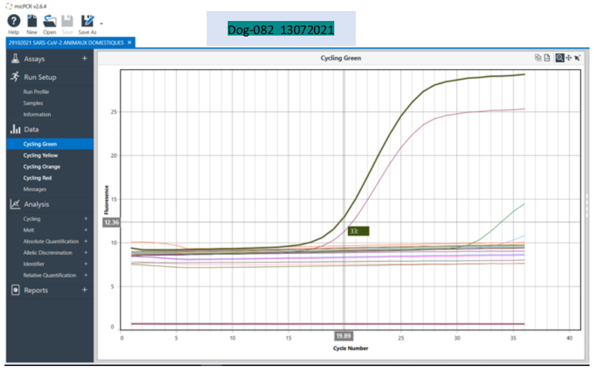
Dog-082 13072021
Cycling Green
Cycle Number

Table S2-Pets's Luminex data

Loc	Sample ID	Net MFI	Net MFI	Dilution factor
		RBD	TRI	
1, A1	Dog-001	87	42	1
1, B1	Dog-002	4527	1596,5	1
1, C1	Dog-004	5374,5	2122	1
1, D1	Dog-006	6809	2521	1
1, E1	Dog-007	598	109	1
1, F1	Dog-008	1554	691	1
1, G1	Dog-010	272,5	78	1
1, H1	Dog-011	332	121	1
1, A2	Dog-012	195	70	1
1, B2	Dog-014	185	80	1
1, C2	Dog-016	125	63	1
1, D2	Dog-017	1285	643,5	1
1, E2	Dog-018	444	43,5	1
1, F2	Dog-019	2469,5	1217,5	1
1, G2	Cat-020	8926	2874	1
1, H2	Cat-021	14017	5795	1
1, A3	Cat-022	1782	479	1
1, B3	Dog-026	147	41	1
1, C3	Dog-028	6273,5	2505	1
1, D3	Dog-029	511	260	1
1, E3	Cat-030	5467,5	2420	1
1, F3	Dog-031	12415	5369	1
1, G3	Dog-032	6420	2118	1
1, H3	Dog-033	93	48,5	1
1, A4	Cat-034	147	115	1
1, B4	Dog-036	118	58	1
1, C4	Dog-037	255	77	1

**1,**	**D4**	Dog-044	247	77,5	1
**1,**	**E4**	Dog-045	163	58	1
**1,**	**F4**	Dog-046	720	114	1
**1,**	**G4**	Dog-047	198	46,5	1
**1,**	**H4**	Dog-048	2984	1034	1
**1,**	**A5**	Dog-049	455	101,5	1
**1,**	**B5**	Dog-050	684	361	1
**1,**	**C5**	Dog-051	1935,5	492,5	1
**1,**	**D5**	Dog-052	4666	1247.5	1
**1,**	**E5**	Dog-054	408,5	325	1
**1,**	**F5**	Dog-055	1604	400	1
**1,**	**G5**	Dog-057	79	36	1
**1,**	**H5**	Dog-058	92	38,5	1
**1,**	**A6**	Cat-059	36	20	1
**1,**	**B6**	Dog-061	396	57	1
**1,**	**C6**	Dog-064	141	96	1
**1,**	**D6**	Dog-065	1846	435	1
**1,**	**E6**	Dog-066	295	80,5	1
**1,**	**F6**	Dog-068	215	86,5	1
**1,**	**G6**	Cat-069	7903	3282	1
**1,**	**H6**	Dog-071	2712	774,5	1
**1,**	**A7**	Dog-072	1778	481	1
**1,**	**B7**	Dog-073	80	64	1
**1,**	**C7**	Dog-074	1878,5	181	1
**1,**	**D7**	Dog-075	4488	1168	1
**1,**	**E7**	Dog-076	96	29	1
**1,**	**F7**	Dog-077	114	33	1
**1,**	**G7**	Dog-078	3792.5	1230	1
**1,**	**H7**	Dog-079	94,5	142	1
**1,**	**A8**	Dog-080	132	65	1
**1,**	**B8**	Dog-081	83	107	1
**1,**	**C8**	Dog-082	10334	4416	1

1,	D8	Dog-083	111	58	1
1,	E8	Dog-085	382,5	58	1
1,	F8	Dog-087	5207	1862,5	1
1,	G8	Dog-088	34	21	1
1,	H8	Dog-089	30	23	1
1,	A9	Dog-090	37,5	19	1
1,	B9	Dog-091	32	18	1
1,	C9	Dog-092	224,5	55	1
1,	D9	Dog-093	299	96	1
1,	E9	Dog-094	3842	628,5	1
1,	F9	CN	52	36	1
1,	G9	CP	28432	830	1

	Loc	Sample ID	Net MFI	Net MFI	Dilution factor
			RBD	Stri	
1,	A1	Dog-095	44	36.5	1
1,	B1	Dog-096	47	39	1
1,	C1	Dog-097	47	42	1
1,	D1	Dog-098	51	43	1
1,	E1	Cat-099	52.5	44	1
1,	F1	Dog-100	49	42	1
1,	G1	Dog-101	45	37.5	1
1,	H1	Dog-102	44	38	1
1,	A2	Cat-103	2820	1589	1
1,	B2	Cat-104	2447	1384.5	1
1,	C2	CN	46	27	1
1,	D2	CP	29105	1025	1
